# Supplementary material for: Synthesis of Chitosan Oligosaccharide-Loaded Glycyrrhetinic Acid Functionalized Mesoporous Silica Nanoparticles and In Vitro Verification of the Treatment of APAP-Induced Liver Injury
Source: Molecules. 2023 May 17;28(10):4147. doi: 10.3390/molecules28104147 (PMC10224134; doi:10.3390/molecules28104147)
Supplement: Supplementary file 1 [file molecules-28-04147-s001.zip › molecules-2334821-supplementary.pdf]

Supporting Information

EXPERIMENTAL SECTION

1. Synthesis of MSN-NH_2-GA

1.1. Preparation of MSNs

A volume of 36 ml of distilled water was added to a round-bottomed flask, a (25 wt%) CTAC solution (24 ml) and TEA (0.09 g) were slowly added, and the solution was magnetically stirred at 70 °C and 150 rpm for 1 h until no bubbles appeared in the solution. Then, 20 ml of 1-octadecene solution containing TEOS (10 v/v%) was slowly added dropwise, the oil bath temperature was set to 70 °C, the rotation speed was set to 150 rpm, and the reaction was continued for 24 h. At this time, the lower layer solution was milky white, and the reaction stopped. When the reaction system returned to normal temperature, the upper layer solution was aspirated with a disposable pipette, and the lower layer of milky white liquid was the target product, which was centrifuged with a high-speed centrifuge at 13,000 rpm for 20 min to obtain a white solid product. The crude product was washed three times with ethanol. Then, the template was washed away and prepared into 40 ml of 1% NaCl methanol solution, and the crude product was washed 3 times in an oil bath at 70 °C under magnetic stirring for 12 h. Finally, the samples were centrifuged at 13,000 rpm with a high-speed centrifuge and washed with ethanol three times to obtain the final product, which was freeze-dried for use.

1.2. Synthesis of amino-functionalized MSNs (MSN-NH_2)

Two hundred micrograms of mesoporous silica MSNs was positioned into a round-bottomed flask, 25 ml of absolute ethanol was added, and after magnetic stirring, 2.0 ml of APTES was added. The temperature was set to 30 °C, the reaction was continued for 24 h, and the solution was then centrifuged at 13000 rpm for 20 min to yield the product MSN-NH_2. MSN-NH_2 was washed 6 times with ethanol and lyophilized with a freeze dryer to obtain the final product. (Figure S1 A)

1.3. Grafting GA onto MSN-NH_2(MSN-NH_2-GA)

First, 0.52 g of NHS, 0.94 g of DCC, and 1.00 g of GA were introduced into a 100-ml spherical round-bottom flask, and 20 ml DMSO was then introduced as a solvent. The mixture was stirred with a magnetic stirrer, 0.50 ml of TEA was slowly added to the reaction system. The reaction was conducted in the dark at 40 °C for 24 h. After the reaction was completed, the heating was stopped, the reaction solution was cooled to room temperature, and the strong product was obtained through suction filtration. The samples were washed three times with 20 ml of ether solution to yield the product GA-NHS, which was lyophilized for use. (Figure S1 B)

GA-NHS and MSN-NH_2 were introduced to anhydrous DMSO, and the reaction solution was stirred at 60 °C for 24 h. The heating was stopped such that the solution cooled to room temperature, and the samples were centrifuged and washed 3 times with anhydrous DMSO and 3 times with ethanol. Glycyrrhetinic acid-modified mesoporous silicon nanoparticles MSN-NH_2-GA were synthesized. (Figure S1 C)

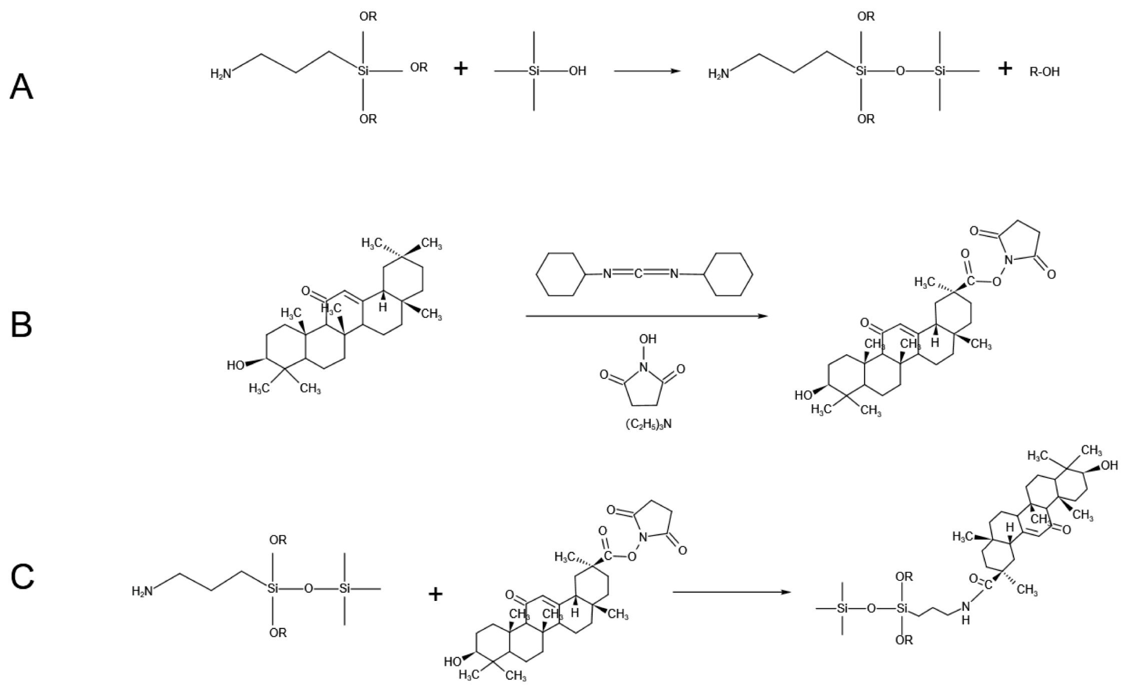

Figure S1. The synthetic route of MSN-NH2 (A), the synthetic route of GA-NHS (B) and the synthetic route of MSN-NH2-GA (C).

2. Biocompatibility experiment of nanoparticles

2.1. Cell culture and determination of nanoparticle cytotoxicity

Culture human normal hepatocytes LO2 in vitro, seed 8000-10000 cells per well in a 96-well plate, observe the cell state with a microscope, after about 10-12 hours of culture; set 0, 200, 400, 800, 1600, 3200 μg/ml MSN and MSN-NH2-GA nanomedicine groups. Each group of nanomedicines was administered according to the preset setting, and the culture was continued for 48 h.

In this study, the CCK-8 method was used to determine the cell viability to evaluate the biocompatibility of MSN and MSN-NH2-GA nanoparticles to cells. From the cell viability results in Figure S2, it can be seen that when the nanoparticle concentration is below 1.6 mg/ml, the Nanoparticles had no significant difference in cell viability, and only when the concentration of nanoparticles increased to 3.2 mg/ml did it have an effect on cell viability. When the concentration is less than 0.8 mg/ml, the nanoparticles have good biocompatibility with cells. The doses of low, medium and high blank nanoparticles were selected as 200 μg/ml, 400 μg/ml and 800 μg/ml, respectively.

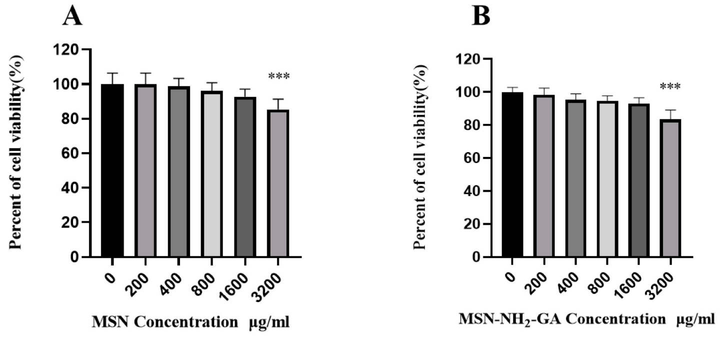

Figure S2. Effect of different MSN and MSN-NH2-GA concentrations on cell viability rate

3. Methodological investigation

3.1. Establishment of analytical method for chitosan oligosaccharide

Under high temperature and acidic conditions, chitosan oligosaccharide was degraded into glucosamine, and under neutral conditions, it reacted with 3,5- dinitrosalicylic acid at high temperature to produce a brownish red product, 3- amino -5-nitrosalicylic acid. In a certain range, there is a good linear relationship between the absorbance of brownish red substance and the content of glucosamine hydrochloride.

The reaction product of glucosamine and DNS, 3- amino -5-nitrosalicylic acid, has a maximum absorption peak at 500 nm, but DNS also has a certain absorption at 500 nm. When the wavelength rises to 540 nm, DNS absorption basically has no absorption. At this time, 3-amino -5-nitrosalicylic acid has a large absorption and DNS interference is the least, so 540 nm is selected as the optimal wavelength for determination.

When the concentration of glucosamine sample is in the range of 100-900 μg/ml, there is a good linear relationship (R^2=0.9991). (Figure S3)

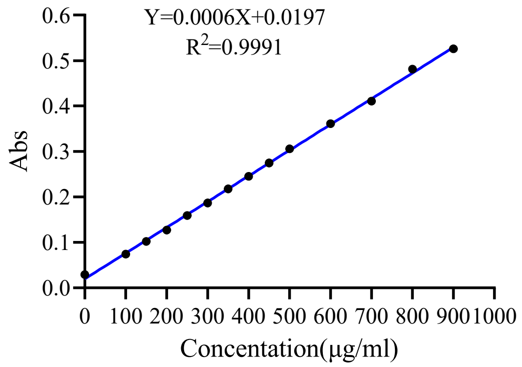

Figure S3. Standard curve of $GluNH_2$ with glucosamine as standard.

3.2. Screening of drug loading conditions

Weigh three parts of nano-carrier MSN-NH_2-GA (10.00 mg each) into a penicillin bottle, add 10.00 ml of PBS phosphate buffer, and ultrasonically disperse it evenly for 10 min in the dark, then weigh 2.50 mg, 5.00 mg and 10.00 mg of COSM to prepare solutions with the ratio of COSM to nanoparticles of 1: 4, 1: 2 and 1: 1. Centrifuge the drug-loaded nano-drug, take 1.00 ml of supernatant, determine the COSM concentration in the supernatant, and subtract the unloaded drug amount from the added drug amount to obtain the drug-loaded quality.

Weigh 10.00 mg of nano-carrier MSN-NH_2-GA into a penicillin bottle, add 10.00 ml of PBS phosphate buffer, ultrasonically disperse it evenly for 10 min in the dark, and add 5.00 mg of COSM. Then, stir on a magnetic stirrer, suck 1.00 ml of supernatant at the time points of 3 h, 6 h, 12 h and 24 h respectively, and add an equal volume of PBS to the reaction system to maintain the system balance, measure the concentration of COSM in the supernatant, and subtract the unloaded dosage from the added dosage to obtain the drug-loaded quality.

The experiment investigated the drug loading rate and encapsulation rate of nano-drug MSN-NH_2-GA with different drug proportions. The results are shown in Table S1. Compared with the three schemes, the drug loading rate of Scheme B and Scheme C is similar, but the encapsulation rate of Scheme B is higher, so Scheme B, that is, the drug loading rate is 28.42± 0.56% and the encapsulation rate is 56.84±1.11%, is selected as the drug loading condition.

Table S1. Investigation of different drug/carrier ratio

	Drug/Carrier ratio (w/w)	COSM (mg)	$MSN-NH_2-GA$ (mg)	EE (%)	LC (%)
A	1:4	2.5	10	13.96±0.45	55.86±1.80
B	1:2	5	10	28.42±0.56	56.84±1.11
C	1:1	10	10	28.63±1.12	28.63±1.12

The influence of drug loading time on drug loading results was investigated. The drug loading rate and encapsulation rate of COSM increased with the increase of time within 0-48 h, and the drug loading rate and encapsulation rate of nanoparticles tended to be stable and basically reached saturation. From Table S2 below, it can be seen that when the drug loading time is 12 h, 24 h and 48 h, there is little difference in the drug loading rate and encapsulation rate of nano-carriers for COSM. In order to improve the efficiency of the experiment, 12 h was chosen as the best drug loading time.

Table S2. Investigation of different drug/carrier ratio

Number	Drug loading time (h)	EE (%)	LC (%)
A	3	11.73±1.03	23.47±2.06
B	6	21.71±1.12	43.43±2.24
C	12	28.36±1.00	56.72±1.99
D	24	28.65±1.12	57.29±2.25
E	48	29.25±1.01	58.49±2.01

4. Modeling and Dosing of Acute Hepatocyte Cell Injury

Cultivate LO2, seed 8000-10000 cells per well in a 96-well plate, observe the cell state with a microscope, after about 10-12 hours of culture; set 0, 2, 4, 6, 8, 10, 12, 14, 16 mM APAP modeling groups. APAP was given to each group according to the preset setting, and the culture was continued for 3, 6, 12, and 24 hours; the cell survival rate was measured by CCK-8 method, and the optimal concentration and time for modeling were determined. According to the results of nanomedicine cytotoxicity, the dosage of COSM@$MSN-NH_2$-GA group: low dose (200 μg/ml), medium dose (400 μg/ml), high dose (800 μg/ml); Group of 6 duplicate holes. The drug loading of COSM@$MSN-NH_2$-GA is 28.36%±1.00%. By equivalent conversion, the dosage of free drug COSM: low dose (56 μg/ml), medium dose (113 μg/ml), high dose (226 μg/ml), 8 duplicate wells in each group, and continued to culture for 12 h after adding COSM drug.

In this study, the optimal concentration and time of APAP for modeling were screened, and the cell viability was measured by CCK-8 method as the evaluation index. It can be seen from the results of cell viability in Figure S4(A) that when the administration time is the same, the viability of LO2 cells is negatively correlated with the APAP concentration. When the APAP concentration is fixed, the modeling time and cell viability are negatively correlated. Taking the cell viability as the ordinate, the cell IC_{50} curve was drawn, and it was found that the inhibition rate was 41.56% ± 3.04% when 10 mmol/L APAP was modeled for 12 h, as shown in Figure S4 (B). According to the IC_{50} principle, the APAP modeling concentration of 10 mmol/L was selected in this experiment, and the modeling time was 12 h.

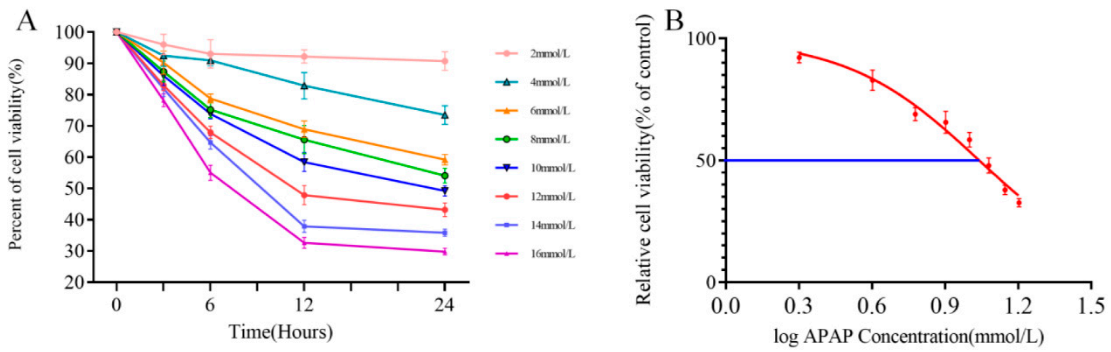

Figure S4. Effect of different modeling concentrations (APAP) and time on cell viability rate (A) and IC_{50} curve (B).
